# First record of *Puerulus mesodontus* Chan, Ma & Chu, 2013 (Crustacea, Decapoda, Achelata, Palinuridae) from south of Java, Indonesia

**DOI:** 10.3897/BDJ.4.e8069

**Published:** 2016-03-07

**Authors:** Yusli Wardiatno, Agus Alim Hakim, Ali Mashar, Nurlisa Alias Butet, Luky Adrianto, Achmad Farajallah

**Affiliations:** ‡ Department of Aquatic Resources Management, Faculty of Fisheries and Marine Science, Bogor Agricultural University, Bogor, Indonesia; § Center for Coastal and Marine Resources Studies, Bogor Agricultural University, Bogor, Indonesia; | Department of Biology, Faculty of Mathematics and Natural Sciences, Bogor Agricultural University, Bogor, Indonesia

**Keywords:** Decapoda, deep-sea spiny lobster, Indian Ocean, new finding, Palabuhanratu bay

## Abstract

**Background:**

Three specimens of *Puerulus mesodontus* Chan, Ma & Chu, 2013 (Crustacea, Decapoda, Achelata, Palinuridae) were collected from Palabuhanratu Bay, southern Java, Indonesia. There is no previous record on the presence of the species in Indonesia. This finding represents the first record of this species in Java, Indonesia, and confirms that the species is present in the Indian Ocean. The morphological characters of the species are described.

**New information:**

This paper contains a new distribution record of a lobster species from Indonesian waters.

## Introduction

The deep-sea spiny lobster genus *Puerulus* Ortmann 1897 lives at depths of 200-700 m in seas of the Indo-West Pacific region facing the Indian Ocean (Holthuis 1991; Chan 1998; Chan et al. 2013). Although the genus is wide in terms of distribution (Chan et al. 2013), it is not of commercial significance (Anrose et al. 2010). However, the genus is economically valued by local communities living on the south coast of Java, Indonesia. Previously, the genus *Puerulus* contained only four species, namely *Puerulus sewelli* Ramadan 1938; *P. velutinus* Holthuis 1963; *P. carinatus* Borradaile 1910; and *P. angulatus* Bate 1888 (Holthuis 1991; Chan 2010). However, the morphological complexity of *P. angulatus*, supported by COI gene sequencing, allowed Chan et al. (2013) to describe five new species, namely *P. quadridentis* Chan, Ma & Chu, 2013; *P. mesodontus* Chan, Ma & Chu, 2013; *P. sericus* Chan, Ma & Chu, 2013; *P. gibbosus* Chan, Ma & Chu, 2013; and *P. richeri* Chan, Ma & Chu, 2013. The new species were not reported from Indonesian waters, but *P. velutinus* and *P. angulatus* were recorded to occur in these waters (Holthuis 1991; Chan et al. 2013). Here, we report, for the first time, that *P. mesodontus* occurs in southern Java, Indonesia.

## Materials and methods

**Figure 1.**
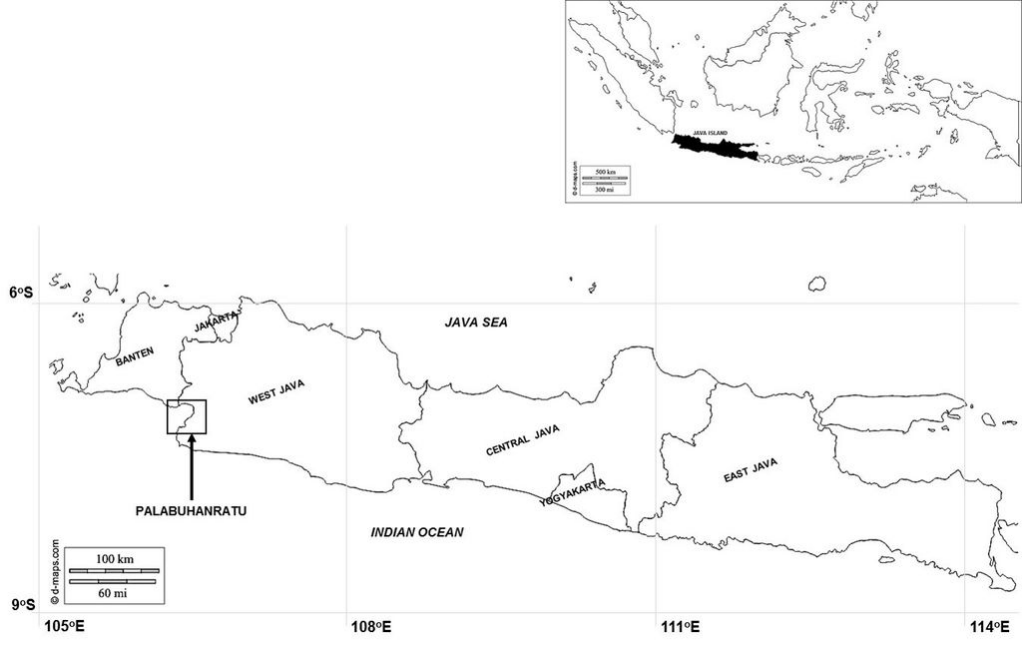
A map of Java Island (insert: map of Indonesia). The open (arrowed) square is Palabuhanratu Bay.

**Figure 2.**
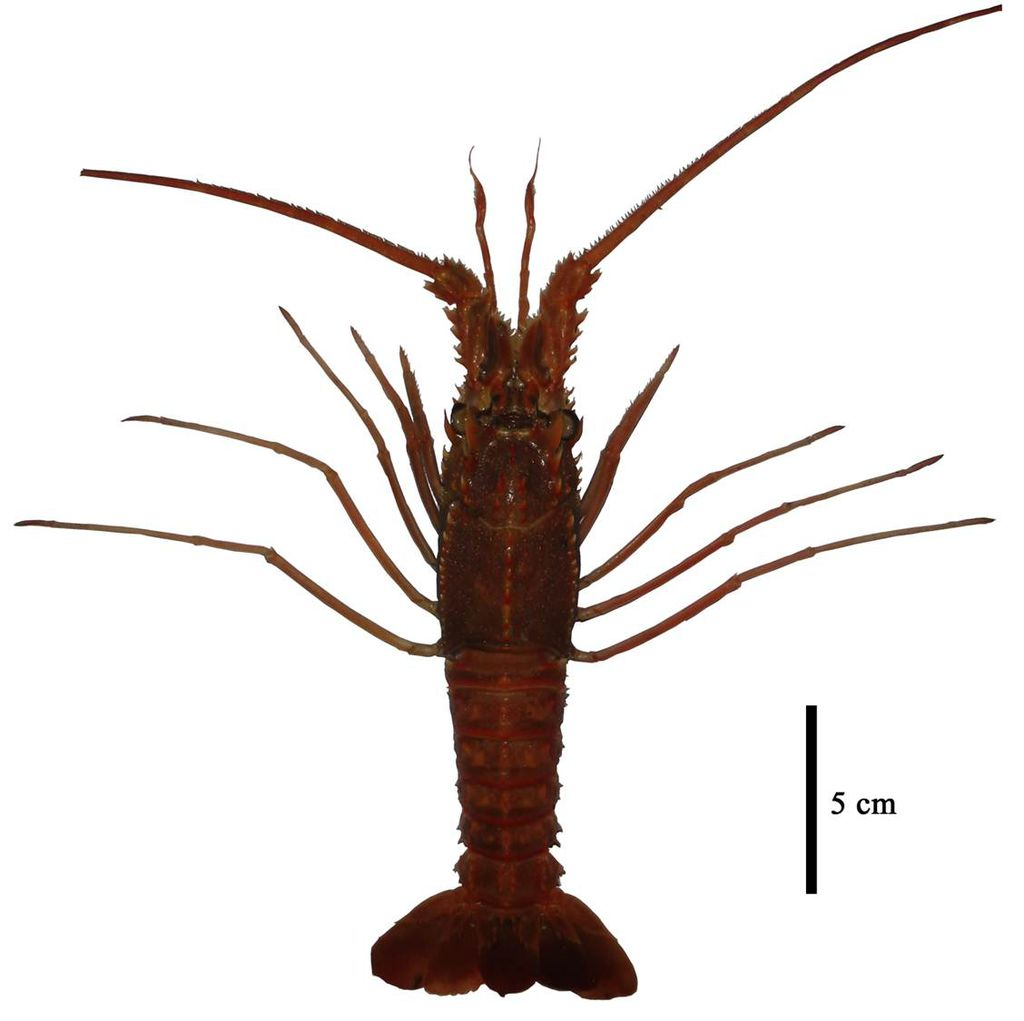
A (female) specimen of *Puerulus mesodontus* collected from Palabuhanratu Bay, southern Java, Indonesia.

Specimens were collected from a fish harbour located in Palabuhanratu Bay, southern Java, Indonesia, in May 2015. The map in Fig. 1 shows the location of the bay. Three specimens were collected, preserved in 96% (v/v) alcohol, and transported to the laboratory for assessment of morphological characters and identification. The specimens were deposited in the collection of the Department of Aquatic Resources Management, Bogor Agricultural University, Indonesia. Identification was based on morphological characters using the taxonomic key and original description from Chan et al. (2013). One specimen is shown in Fig. 2.

## Taxon treatments

### Puerulus mesodontus

Chan, Ma & Chu, 2013

#### Materials

- **Type status:** Other material. **Occurrence:** recordedBy: Yusli Wardiatno, Agus Alim Hakim, Ali Mashar, Nurlisa Alias Butet, Luky Adrianto, Achmad Farajallah; individualCount: 3; preparations: specimen stored in ethanol 96%; otherCatalogNumbers: PMP01, PMP02, PMP03; **Taxon:** scientificNameID: *Puerulus mesodontus* Chan, Ma & Chu, 2013; scientificName: Puerulus mesodontus; kingdom: Animalia; phylum: Arthropoda; class: Malacostraca; order: Decapoda; family: Palinuridae; genus: Puerulus; specificEpithet: mesodontus; scientificNameAuthorship: Chan, Ma & Chu 2013; **Location:** waterBody: Indian Ocean; stateProvince: West Java; locality: Palabuhanratu Bay; verbatimLocality: Palabuhanratu; maximumDepthInMeters: 20; **Identification:** identifiedBy: Agus Alim Hakim; dateIdentified: 2015-12-22; identificationRemarks: identified by morphology; identificationQualifier: cf.; **Event:** samplingProtocol: local fishing boat and net; eventDate: 2015-05-17; eventTime: unrecorded; habitat: sandy bottom; **Record Level:** language: ina; basisOfRecord: PreservedSpecimen

#### Description

Body moderately pubescent. Carapace similar in length to abdominal somites I-V; surfaces mostly covered with spinules and sharp granules. Supraorbital horn just overreaching the eye and extending to a position approximating the basal one-third of the antennular plate; dorsal margin almost straight, and smooth; followed by a row of three regularly spaced teeth decreasing sharply in size posteriorly; third tooth always prominent. Eye more long than broad (Fig. 3a, b).Postorbital spinules well-developed. Two rows of cervical spines converging anteriorly into a well-developed median spine. Three gastric teeth; the first tooth generally smaller or the third tooth generally larger; sometimes all gastric teeth similar in size or occasionally the first tooth larger; the base of the third tooth less than 1.5-fold wider than the base of the first tooth; the third tooth distinctly distant from the two anterior teeth (Figure 3b). Pereopod V not chelate in males, but chelate in females (Fig. 3c, d). The merus of maxilliped III with an anterodorsal spine (Fig. 3e). The abdomen with a raised granular-to-lobular surface, but the granules not forming distinct rows (Fig. 3f).

## Discussion

Most works on Indonesian lobsters have focused on the economically important species, such as diversity, distribution and genetic population of *Panulirus penicillatus* Olivier 1791 (Chow et al. 2011, Kalih 2012, Abdullah et al. 2014), first record occurrence and sexual dimprphism of *Linuparus somniosus* Berry and George 1972 (Wowor 1999), size distribution, length-weight relationship, condition factor, and sex ratio of *Panulirus versicolor* Latreille 1804 (Ongkers et al. 2014), and the distribution of *Panulirus homarus (Linnaeus 1758), P. longipes* (Milne-Edwards 1868), *P. ornatus* (Fabricius 1798), and *Parribacus antarticus* (Lund 1793) in Lombok island and its surrounding waters (Kalih 2012). In this paper we present the first record of *Puerulus mesodontus* from Pelabuhanratu Bay, southern Java. This is also the first record of the species in Indonesia. Chan et al. (2013) describes the distribution of *P. mesodontus* in the western Pacific as Japan, Taiwan, the Philippines, Papua New Guinea, the Solomon Islands, Vanuatu, New Caledonia, and Fiji.

This finding adds to new records of Indonesian crustaceans, which now include *Albunea symmysta* (Linnaeus, 1758) (Mashar et al. 2015), *Hippa marmorata* (Hombron and Jacquinot 1846) (Wardiatno et al. 2015), and *Hippa adactyla* Fabricius 1787 (Ardika et al. 2015). The finding emphasises the fact that Indonesian waters constitute a hotspot of Asian aquatic biodiversity. Future research on the species should focus on distribution, population dynamics, and genetic diversity.

## Supplementary Material

XML Treatment for Puerulus mesodontus

## Figures and Tables

**Figure 3a.**
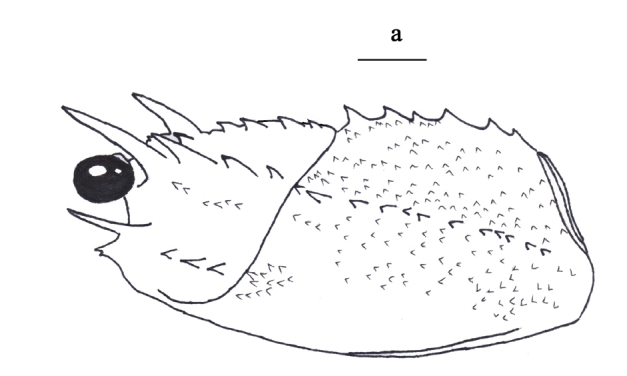
carapace and eye, lateral view.

**Figure 3b.**
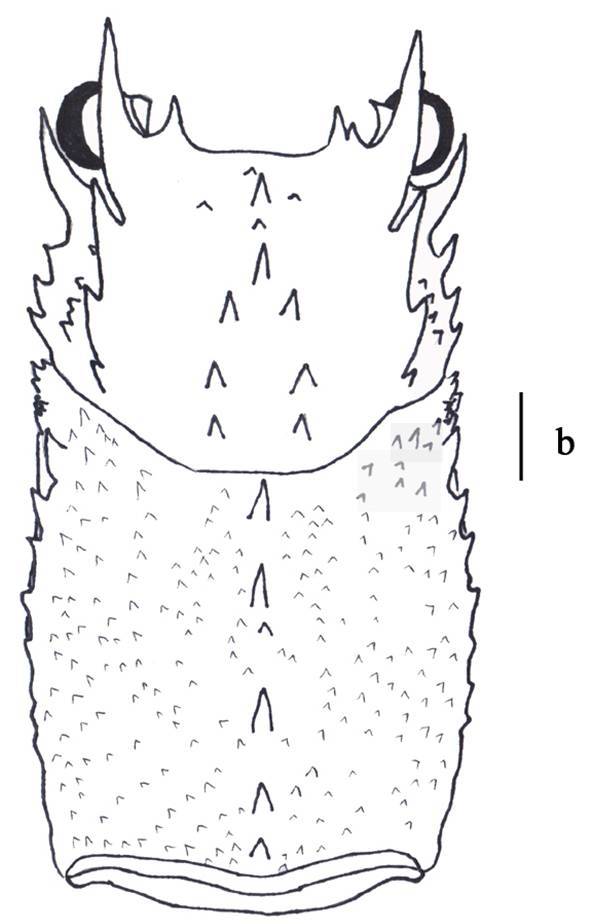
carapace, dorsal view.

**Figure 3c.**
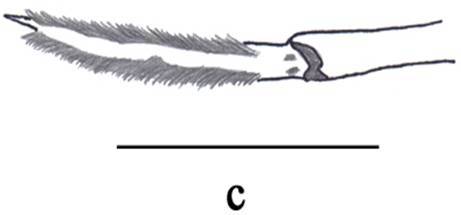
male, dactylus and distal part of propodus of left pereopod V.

**Figure 3d.**
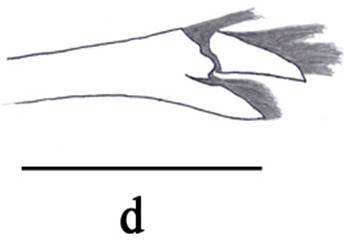
female, dactylus and distal part of propodus of left pereopod V.

**Figure 3e.**
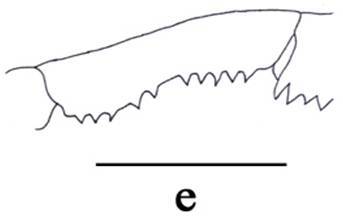
left merus of maxilliped III, lateral view.

**Figure 3f.**
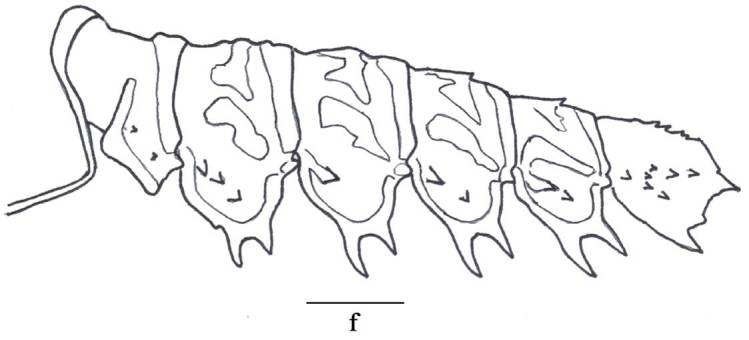
posterior carapace and abdominal somites I to VI, lateral view.
